# Diagnosis and Management of Gastrointestinal Foreign Bodies

**DOI:** 10.1155/2016/5692650

**Published:** 2016-10-16

**Authors:** Kenya Kamimura, David C. Evans, Ken-ichi Mizuno, Juan E. Sola

**Affiliations:** ^1^Division of Gastroenterology and Hepatology, Graduate School of Medical and Dental Sciences, Niigata University, 1-757 Asahimachido-ri, Chuo-ku, Niigata 9518510, Japan; ^2^Department of Surgery, The Ohio State University, 395 W. 12th Avenue, Columbus, OH 43210, USA; ^3^Division of Pediatric Surgery, University of Miami, 1611 NW 12th Avenue, Miami, FL 33136, USA

Gastrointestinal foreign bodies (GIFBs) are common conditions that often present as emergencies affecting all generations worldwide [1–3]. Therefore, knowledge and experiences regarding GIFBs should be shared for prompt diagnoses followed by expert clinical management. For example, while children commonly ingest coins, toys, batteries, and magnets, adults may ingest meat, bones, rings, and so forth, while eating. In addition, in the elderly, the partial denture has also become a common object of ingestion. Patients with GIFBs show symptoms of nausea, vomiting, dysphagia, chest pain, and difficulty in eating food or drinking water. With the development of endoscopic technology and devices, the removal of these materials has become less invasive, and successful removal rates have increased. Therefore, this special issue provides a current review of GIFBs and the latest therapeutic methods, which will allow physicians to make correct treatment decisions to save patients' lives in the emergency department.

M. Bekkerman et al. have outlined the current status of GIFBs, including symptoms, patient characteristics, clinical courses of patients with GIFBs, diagnostic modalities, and endoscopic management approaches. They have also reviewed the literature reported to date, concluding that an endoscopic approach to GIFB removal is both safe and effective.

The next section covers the characteristics of GIFBs in children and adults, including the elderly, through a review (“*Endoscopic Management of Ingested Foreign Bodies in the Gastrointestinal Tract: A Review of the Literature*” by M. Bekkerman et al.) and an original article (“*Foreign Bodies Ingestion in Children: Experience of 61 Cases in a Pediatric Gastroenterology Unit from Romania*” by S. Diaconescu et al.). While food boluses (e.g., meat), fish bones, chicken bones, dentures, and so forth are common GIFBs in adults; coins, button batteries, toys, and so forth are major GIFBs in children. S. Diaconescu et al. reported 61 childhood cases in Romania who were unable to be successfully treated with an endoscopic procedure; these authors reported complications, such as tracheal-esophageal fistula, caused by a battery.

For a deeper understanding of GIFBs in adult cases, one of the etiologies of food bolus impaction in young adults has been analyzed in an original article (“*Prevalence of Eosinophilic Esophagitis and Lymphocytic Esophagitis in Adults with Esophageal Food Bolus Impaction*” by K. Truskaite et al.). K. Truskaite et al. showed that eosinophilic esophagitis was the leading cause of food bolus impaction in a review of 185 cases in whom biopsies were performed. These authors reported that approximately 40% of these cases were complicated with eosinophilic esophagitis, concluding that the esophageal biopsies in patients with food bolus impaction were essential to the diagnosis of eosinophilic esophagitis and the management of the cases thereafter. The state-of-the-art endoscopic technology for both general GIFB removal and ingested dentures specifically is also reviewed (“*Endoscopic Management of Ingested Foreign Bodies in the Gastrointestinal Tract: A Review of the Literature*” by M. Bekkerman et al. and “*Endoscopic Removal of Ingested Dentures and Dental Instruments: A Retrospective Analysis*” by K. Mizuno et al.). K. Mizuno et al. reported the efficacy and safety of an endoscopic removal procedure for ingested dentures and dental instruments, examining 29 cases at their institute. These authors concluded that immediate intervention and appropriate device selection are essential for ingested dentures. In addition, an original research article by K. Ogawa et al. reviewed the etiology of and general background on bezoars, which are common worldwide. These authors describe the successful therapeutic effects of combination therapy of dissolution, using a carbonated liquid and an endoscopic procedure (“*The Combination Therapy of Dissolution Using Carbonated Liquid and Endoscopic Procedure for Nezoars: Pragmatical and Clinical Review*” by K. Ogawa et al.).

These new therapeutic strategies for GIFB removal will directly support physicians who treat affected patients. In addition, this information will further improve the procedures used, lead to the development of new devices for new therapeutic methods, and establish a mechanical model for medical training.

In summary, this special issue will provide the current understanding of GIFBs and describe recent progress in the therapeutic methods that are used in clinical practice.

*Kenya Kamimura* *Kenya Kamimura* *David C. Evans* *David C. Evans* *Ken-ichi Mizuno* *Ken-ichi Mizuno* *Juan E. Sola* *Juan E. Sola*
